# Tetraspanin Cd9b plays a role in fertility in zebrafish

**DOI:** 10.1371/journal.pone.0277274

**Published:** 2022-11-10

**Authors:** Sarah Greaves, Katherine S. Marsay, Peter N. Monk, Henry Roehl, Lynda J. Partridge

**Affiliations:** 1 School of Biosciences, University of Sheffield, Sheffield, United Kingdom; 2 Institute of Molecular and Cell Biology, A*STAR, Singapore, Singapore; 3 Department of Infection, Immunity and Cardiovascular Science, University of Sheffield, Sheffield, United Kingdom; University of Colorado Boulder, UNITED STATES

## Abstract

In mice, CD9 expression on the egg is required for efficient sperm-egg fusion and no effects on ovulation or male fertility are observed in CD9 null animals. Here we show that *cd9b* knockout zebrafish also appear to have fertility defects. In contrast to mice, fewer eggs were laid by *cd9b* knockout zebrafish pairs and, of the eggs laid, a lower percentage were fertilised. These effects could not be linked to primordial germ cell numbers or migration as these were not altered in the *cd9b* mutants. The decrease in egg numbers could be rescued by exchanging either *cd9b* knockout partner, male or female, for a wildtype partner. However, the fertilisation defect was only rescued by crossing a *cd9b* knockout female with a wildtype male. To exclude effects of mating behaviour we analysed clutch size and fertilisation using *in vitro* fertilisation techniques. Number of eggs and fertilisation rates were significantly reduced in the *cd9b* mutants suggesting the fertility defects are not solely due to courtship behaviours. Our results indicate that CD9 plays a more complex role in fish fertility than in mammals, with effects in both males and females.

## Introduction

CD9 is a member of the tetraspanin superfamily of proteins that function as organisers of other membrane proteins [1]. CD9 is involved in a wide range of cell functions, including adhesion, motility, signalling and cell fusion [2]. Knockout (KO) of CD9 in female mice results in infertility due to a defect in sperm/oocyte fusion (reviewed in [3]). In a mechanism that appears conserved in mammals, CD9 is suggested to partner Juno, the egg receptor for sperm ligand Izumo1, thus facilitating the formation of adhesion sites prior to fusion [4]. CD9 concentrates at the interaction site on the oocyte in response to sperm oscillations immediately before fusion [5]. Whilst male CD9 KO mice appear fertile, CD9 is expressed on mouse sperm and male germline stem cells [6, 7] and is present at various stages of spermatogenesis, suggesting a role in this process [8].

Tetraspanins are widely expressed in teleosts [9] but there are no reports of roles in fish fertility. Several tetraspanins have roles during zebrafish development, including pigment cell interactions [10], hatching [11], vascularisation [12, 13] migrasome formation [14] and primordium migration [15]. However, the role of Cd9 in fertilisation has not yet been investigated. There are two paralogues of cd9 in zebrafish (cd9a and cd9b), which have 63% amino acid identity and similar mRNA expression patterns [15].

In this report, we investigate the role of Cd9 in zebrafish fertility. Two zebrafish *cd9b* alleles were used, and homozygous in-crosses of both alleles exhibited defects in fertilisation rates. The number of eggs produced per female (clutch size) was also significantly reduced. The defect in fertilisation was not further exacerbated by the additional KO of the paralogue, *cd9a*. Reduced clutch size could be rescued by crossing either *cd9b* KO male or female fish with a wildtype (WT) partner. In contrast, reduced fertilisation was only rescuable by crossing a KO female with a WT male. Our results indicate that Cd9 plays a more complex role in fish fertility than in mammals, with effects on both male and female fertility.

## Methods and materials

### Zebrafish maintenance

Adult wildtype AB zebrafish (WT) and *cd9a/b/dKO* mutants were housed and bred in a regulated 14:10 hour light: dark cycle under UK Home Office project licence 40/3459 in Bateson Centre aquaria at the University of Sheffield or project licence IACUC 140924 in the Singapore IMCB zebrafish facility. Zebrafish were raised under the standard conditions at 28°C [16].

### Zebrafish mutant production

*cd9b* mutants were created from WT embryos using transcription activator-like effector nucleases (TALEN) and maintained on an WT background. TALENs (ZGene Biotech Inc., Taiwan) were provided in a pZGB4L vector, targeting the *cd9b* sequence 5’ ttgctctttatcttca 3’. Two frameshift mutants, c.46del (*cd9b*^*is16*^ allele) or c.42_49del (*cd9b*^*pg15*^ allele) were selected that caused premature termination in the second transmembrane domain or just after the first transmembrane domain respectively (S1 Fig) (previously described by Marsay et al., 2021). *cd9a* mutants were created by Marsay et al., 2021 using CRISPR/Cas9. An indel mutation deleting 4bp and inserting 8bp (c.180_187delinsTCGCTATTGTAT; *cd9a*^*la61*^) generated a frameshift mutation resulting in a premature stop codon in exon 3, which was predicted to truncate the protein before the large extracellular domain. *cd9* dKO mutants were created by injecting the *cd9a* gRNA and Cas9 RNA into *cd9b*^*pg15*^ embryos. These fish were screened for germline transmission by sequencing and backcrossed to *cd9b*^*pg15*^ mutants. Heterozygous fish of the same genotype were in-crossed and adult F2 fish were genotyped to identify homozygous *cd9b*^*pg15*^*; cd9a*^*la61*^ (*cd9* dKO).

### Embryo collection and analysis

Adult zebrafish male/female pairs were placed in plastic breeding tanks overnight, separated by a divider. The wildtype and mutants were not siblings but fish of similar sizes were paired together when conducting pair mating experiments. All AB fish used were born within the same month and the *cd9b* mutants were born within 2 months of the AB fish used. The divider was removed the following morning after the lights came on and spontaneous spawning occurred. Embryos were collected every 20 min and collection time was recorded. Zebrafish pairs were allowed to spawn until no more embryos were produced and the number of embryos produced by each pair was recorded. Dead eggs (opaque eggs) were counted and removed on collection and fertilisation was assessed four hours post collection. Embryos that presented a well-developed blastodisc were counted as fertilised. It is known that egg laying is highly variable and so repeats were carried out over several weeks to ensure that the results were robust. Details of repeats are described in each figure legend. In addition, the experiments were conducted in the same environment, with the same protocol and the same equipment to try to minimise any environmental variation or influence.

### Probe synthesis for *in situ* hybridisation

*vasa* cDNA was provided by H. Knaut (NYU Medical Center and School of Medicine, USA) in a pBS+ cloning vector. The vector conferred ampicillin resistance and contained M13 primer binding sites flanking the *vasa* cDNA. *vasa* cDNA containing plasmid was transformed into NEB 10-beta competent *E*.*coli*, and purified using a Miniprep kit (Qiagen, UK). The DNA template for the *vasa* RNA probe was then produced using a standard PCR protocol with M13 primers (Forward: 5’gtaaaacgcggccagt3’, Reverse: 5’ggaaacagctatgaccatg 3’), and purified using a 50 kDal centrifugal filter unit (Amicon, UK). Anti-sense RNA probes were transcribed from the DNA template using digoxigenin (DIG)-11-UTP Labelling Mix (Roche, UK), cleaned using spin filters (Sigma-Aldrich) and eluted into RNA-later (Sigma-Aldrich, UK) before storing at -20°C.

### *In situ* hybridisation

Embryos were raised at 28°C in petri dishes containing E3 solution. The E3 was changed daily and any dead embryos removed. At 30–32 hours post fertilisation (hpf), embryos were anaesthetised using tricaine, dechorionated and then fixed using 4% (w/v) paraformaldehyde (PFA; Sigma-Aldrich, UK) in PBS. The fixed embryos were left overnight at 4°C in 4% PFA before being washed twice with PBS/0.05% (v/v) Tween 20 (PBST) the following morning. Embryos were then put through a MeOH/PBS series using 30%, 60% and 100% (v/v) MeOH before being stored in 100% MeOH (Sigma-Aldrich) at -20°C. *In situ* hybridisation was carried out as described (Thisse and Thisse, 2008), except for the embryo digestion with proteinase K, for which 30–32 hpf embryos were digested with 10 mg/ml proteinase K at 20°C for 22 min. The protocol was performed with embryos in 1.5 ml microfuge tubes for the first two days, after which they were placed in 12-well plates for staining before transferring back to microfuge tubes for storage. Stained embryos were stored in the dark in 80% (v/v) glycerol.

### qRT-PCR

Quantitative reverse transcription-PCR (qRT-PCR) was conducted using the Sigma S5193 kit and run on a Stratagene qPCR machine using MXPro software. All reactions were set up with 1 μL of 7.5 μM primer, 2.8 μL 25 mM MgCl_2_, 0.2 μL ROX reference dye, 10 μL SYBR Green ready mix, (Jumpstart) and 5 μL 1:20 cDNA. All reactions followed the following thermal cycle; 3 minutes initial denaturation at 95°C, 40 cycles of 15 seconds at 95°C, 15 seconds at 57°C and 20 seconds at 72°C, then finally 1 minute at 95°C, 30 seconds at 55°C and 30 seconds at 95°C. Primers were first tested to ensure they did not produce primer dimers or other non-specific products by checking for a sharp peak in the melting curve (S2 Fig). Primers used were β-actin2 F 5’- ggacctgtatgccaacactg-3’, β-actin2 R 5’- tgatctccttctgcatcctg-3’, *cd9b* F 5’-gaacccgtgacatcgtgtaa-3’ and *cd9b* R 5’- tacaacaggacaaccactcg-3’. The fold expression was calculated by initially normalising the expression of *cd9b* to the control gene, *β-actin-2*, and then differences in fold expression between mutants and WT were calculated by normalising the mutant expression to the wild type.

### Primordial germ cell (PGC) assays

PGC were stained using a *vasa in situ* hybridisation and then embryos were imaged in 80% glycerol using a microscope mounted camera and a 5x or 10x objective. The number of PGCs was counted across the whole embryo and PGC migration was analysed by measuring the distance between the most anterior and posterior PGCs, with the measurement following the body axis. Measurements were taken using Image J software.

### *In vitro* fertilisation (IVF)

Adult zebrafish were paired, as described above, 4 days before the IVF procedure and then transferred back to their normal tanks. The fish were then paired again in the afternoon before the IVF procedure. The following morning, fish of the same genotype were placed together in larger tanks as zebrafish will not normally lay when grouped. Individual fish were then anaesthetised using tricaine and dried before gamete extraction. Sperm was extracted from male fish using suction through 10 μl capillaries (Hirschmann Laborgeräte GmbH, Germany), whereas females were gently pressed on the abdomen to release eggs. Gametes from a single pair of individuals were combined and incubated for 30 sec before adding 750 μl aquarium water and incubating for a further 2 min. 9 ml of aquarium water was then added and the gametes incubated for 4 hr at 28°C. The numbers of fertilised and unfertilised eggs were then assessed. Dead eggs were immediately discarded after extraction from the females and therefore not included in the analysis.

### Statistics

Data distribution was first assessed for normality using a D’Agostino-Pearson omnibus K2 normality test on the experimental residuals, as well as creating a histogram of residuals. For normally distributed data, an ANOVA with Dunnet’s or Holms-Sidak multiple comparisons tests were used. For non-normally distributed data non-parametric tests, the Mann-Whitney U test or Kruskal-Wallis with Dunn’s multiple comparisons test, were used.

## Results and discussion

**Fig 1:**
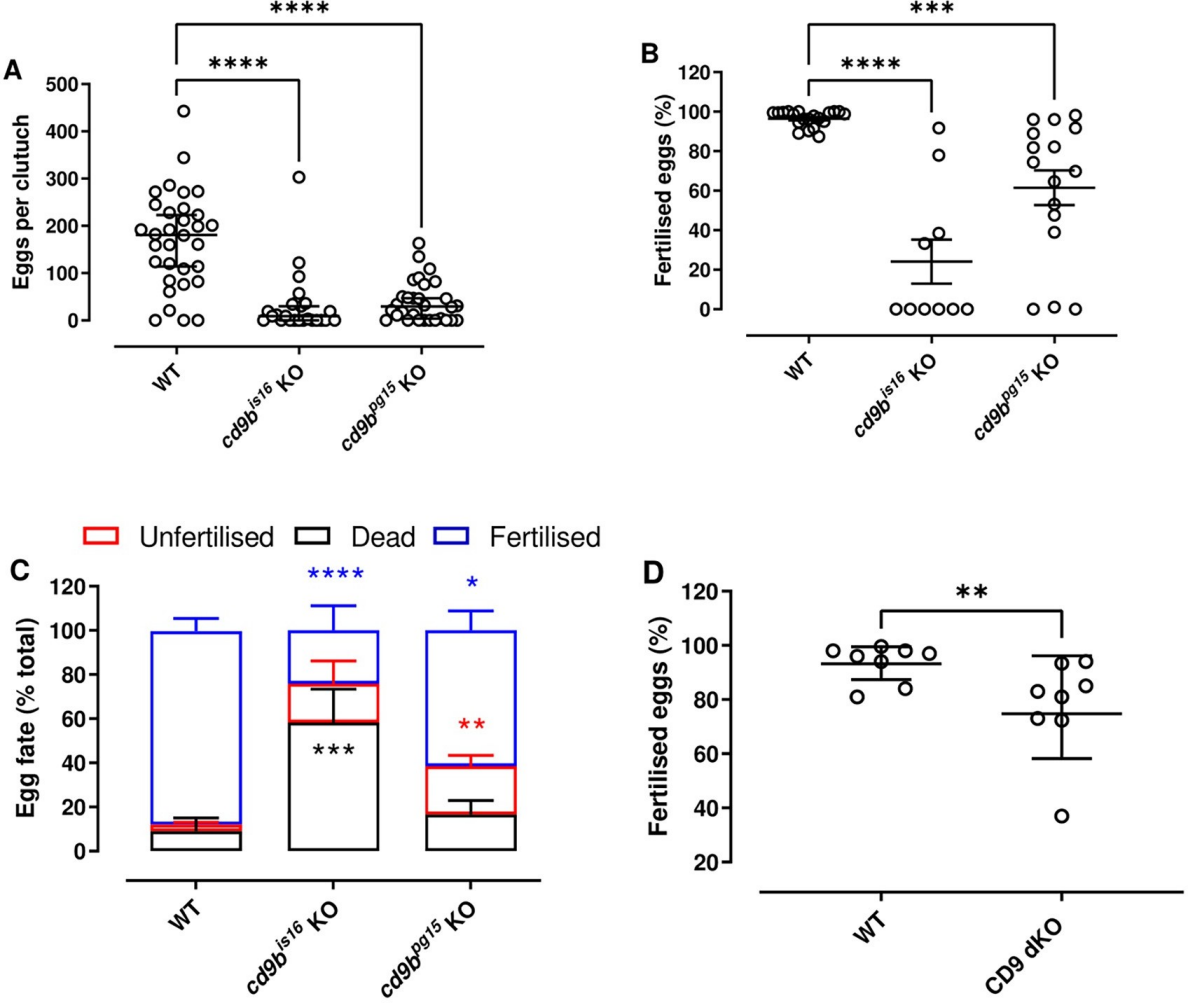
*cd9b* mutant pairs lay smaller clutches of eggs with significantly fewer fertilised eggs. A significant decrease in the total number of eggs laid per pair, including fertilised, dead and unfertilised eggs, is seen with *cd9b* mutant pairs (A). The percentage of fertilised eggs is significantly decreased in clutches from *cd9b* mutant pairs (B). Given the significant decrease in fertilised eggs seen in (B), it can be inferred that there is a significant increase in the percentage of dead and unfertilised eggs per clutch from *cd9b* mutants. This increase is apparent in (C), where the average breakdown of clutches from WT and *cd9b* mutant pairs is shown. The additional loss of *cd9a* in *cd9* dKOs did not result in a further decrease in the fertilisation rate (D). Data from 5 experimental repeats carried out over several weeks. (A), (B) and (C) represent pooled data from 5 experimental repeats. A Kruskal-Wallis test with Dunn’s multiple comparisons test where p<0.05 was performed on (A). An ANOVA with Dunnett’s multiple comparisons test was performed on (B, C) after removal of definitive outliers (3 outliers, ROUT, q = 0.1) to give normally distributed data (p<0.05). n = minimum 25 pairs (A), minimum 10 pairs (B, C) per genotype. Only pairs that laid eggs were counted in (B) and (C), whereas all pairs were counted in (A). (D) represents 8 separate matings, with a Mann-Whitney U test of significance. Significance of difference from WT control: **** p<0.001; *** p<0.005; ** p<0.01; * p<0.05; ns = not significant.

To test the involvement of Cd9b in zebrafish fertility, we used two alleles of Cd9b, *cd9b*^*is16*^ and *cd9b*^*pg15*^. Both alleles were selected as they caused frameshift mutations in the N-terminus and premature termination in or before the second transmembrane domain (S1 Fig). The homozygous mutant KO fish appeared to develop normally. However, when in-crossed, both the number of eggs per clutch (Fig 1A) and the fertilisation rate of the eggs produced were significantly reduced compared to WT (Fig 1B). While the number of eggs produced appear similarly reduced in both alleles (Fig 1A), the extent of reduction in fertility differed dramatically between the two alleles with *cd9b*^*is16*^ KO mutant pairs producing a markedly lower percentage of fertilised eggs (Fig 1B). The loss of fecundity in both alleles was surprising because the KO of CD9 in mice affects only the fertilisation of ova and not their production [17]. When the fate of the zebrafish eggs was analysed in more detail, the *cd9b*^*is16*^ KOs produced a significantly higher percentage of eggs that were dead at the time of embryo collection (Fig 1C). Dead eggs are opaque and are easily identified. However, this significant increase in the percentage of dead eggs was not replicated in the *cd9b*^*pg15*^ KO line, which produced a significant number of live but unfertilised eggs. (Embryos that presented a well-developed blastodisc after 3 hours were counted as fertilised). The difference in severity between the alleles could be due to the differences in mutations. While both mutations occur in similar codons (15 and 16), the consequent frameshift causes slightly more aberrant amino acids in the *cd9b*^*is16*^ KOs before a stop codon is created (46 in contrast to 22 in *cd9b*^*pg15*^ KO). While ISH and qPCR results showed a downregulation of mRNA suggesting nonsense mediated decay was occurring (S2, S3 Figs) [15], there could be differences in the residual function of the mutated protein. It is known, for example, that synthetic cell permeable peptides corresponding to the termini of CD9 show cellular activity [18]. There could also be differences in the amount of genetic compensation induced in either mutant [19]. Tetraspanins are known to have high levels of redundancy due to high structural relation, complementary roles and similar partner proteins. This increases the likelihood that there is some compensation happening in the *cd9b* KOs. The most likely candidate is *cd9a* as they share high protein identity and similar mRNA expression patterns to *cd9b* [15].

To determine if deletion of both paralogs would result in a complete loss of fertility as seen in the CD9 KO mouse, *cd9a* was knocked out in the *cd9b*^*pg15*^ background. ISH results show downregulation of *cd9b* and *cd9a* mRNA suggesting nonsense mediated decay was occurring [15]. Interestingly, the fertilisation rate of the double KO line was very similar to the *cd9b*^*pg15*^ KO line (Fig 1D), suggesting that only Cd9b is involved in egg production and fertilisation.

**Fig 2:**
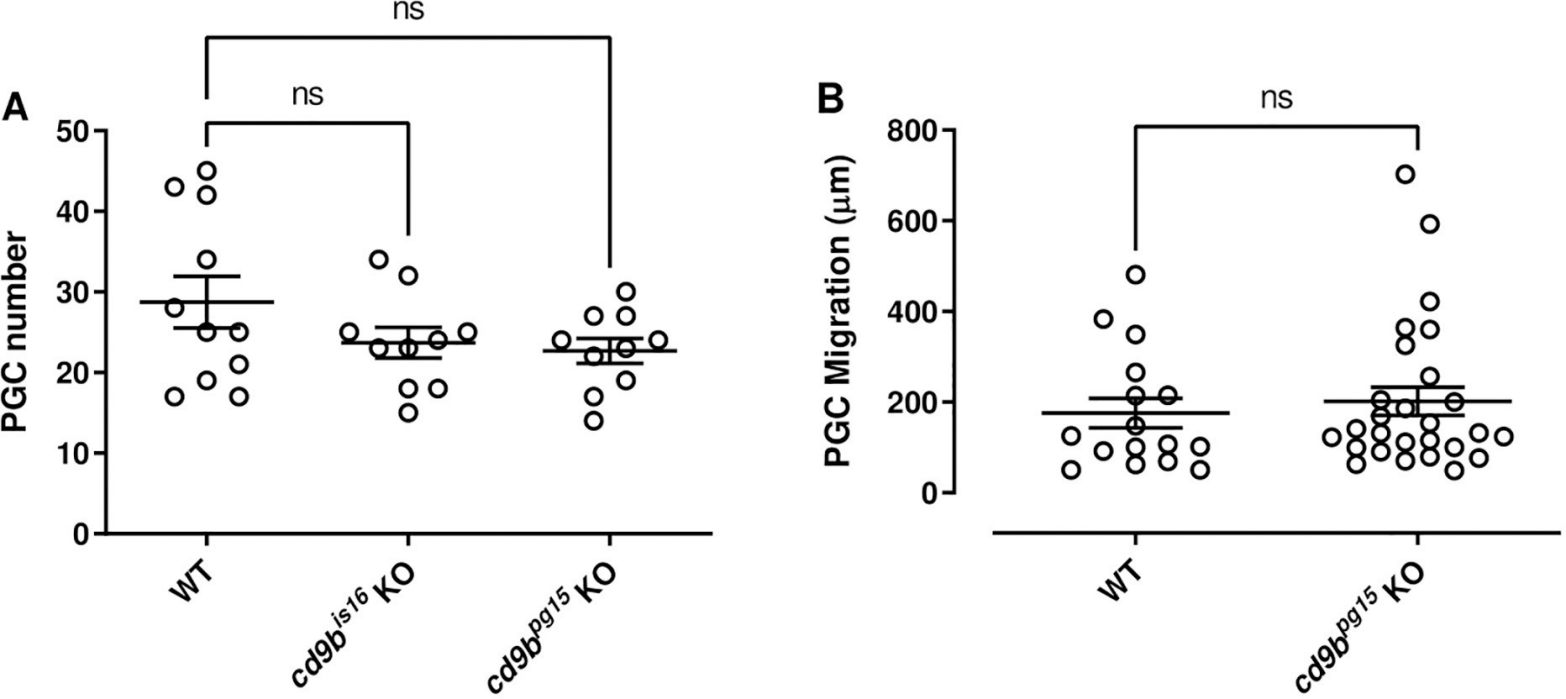
*cd9b* homozygous mutants have the same number of PGC per embryo at 32 hpf and migration to the gonadal ridge is not affected in *cd9b*^*pg15*^ mutants at 30 hpf. (A) There is no significant difference in the number of PGCs in WT and *cd9b* mutant embryos at 32 hpf. PGCs were visualised using a *vasa* ISH on 32 hpf embryos and analysed using an ANOVA with Dunnett’s multiple comparisons test on n = minimum 10 embryos per genotype. (B) *vasa* ISH on 30 hpf WT and *cd9b*^*pg15*^ embryos. PGC migration efficiency was analysed by looking at the distance between the most anterior and posterior PGC, which should have reached the gonadal ridge by 30 hpf. No significant difference was seen between WT and *cd9b* mutants as shown by a Mann-Whitney U test. n = at least 13 individual embryos. Data from a single experiment.

We then investigated primordial germ cell (PGC) behaviour, to determine if reduced numbers or a delayed migration could result in lowered egg production [20]. However, both *cd9b* KO lines have the same number of PGC as WT fish (Fig 2A) and migration to the gonadal ridge during early development was not altered in *cd9b* mutants (Fig 2B). These results suggest that the number and migration of PGCs does not play a causative role in the reduction of fecundity and fertility seen with *cd9b* mutant pairs. These preliminary results do not, however, eliminate the possibility that mutations in *cd9b* could impact gonad development and morphology in later development or lead to impaired gametogenesis in mature gonads. Future experiments to elucidate possible roles for Cd9b in these processes could include histological analysis of the gonads at stages throughout development, analysis of gametogenesis and reproductive hormones such as follicular stimulating hormone and luteinizing hormone in sexually mature zebrafish, as well as investigating the expression of genes known to play a role in gonad development (e.g. *ar*, *cyp11c1*, *cyp17a1*, *fancl*, *foxl2*, *hsf5*, *piwil1*, *piwil2)* [21].

It would also be beneficial to undertake an analysis of *cd9b* expression to determine whether *cd9b* is expressed in the germs cells or the gonads. If *cd9b* is expressed in the germ cells, it would be interesting to study *cd9b* expression at different stages of oogenesis and spermatogenesis, given that CD9 has been shown to be expressed on murine oocytes and spermatogonial stem cells, as well as throughout the majority of spermatogenesis in mice [2, 22–24].

**Fig 3:**
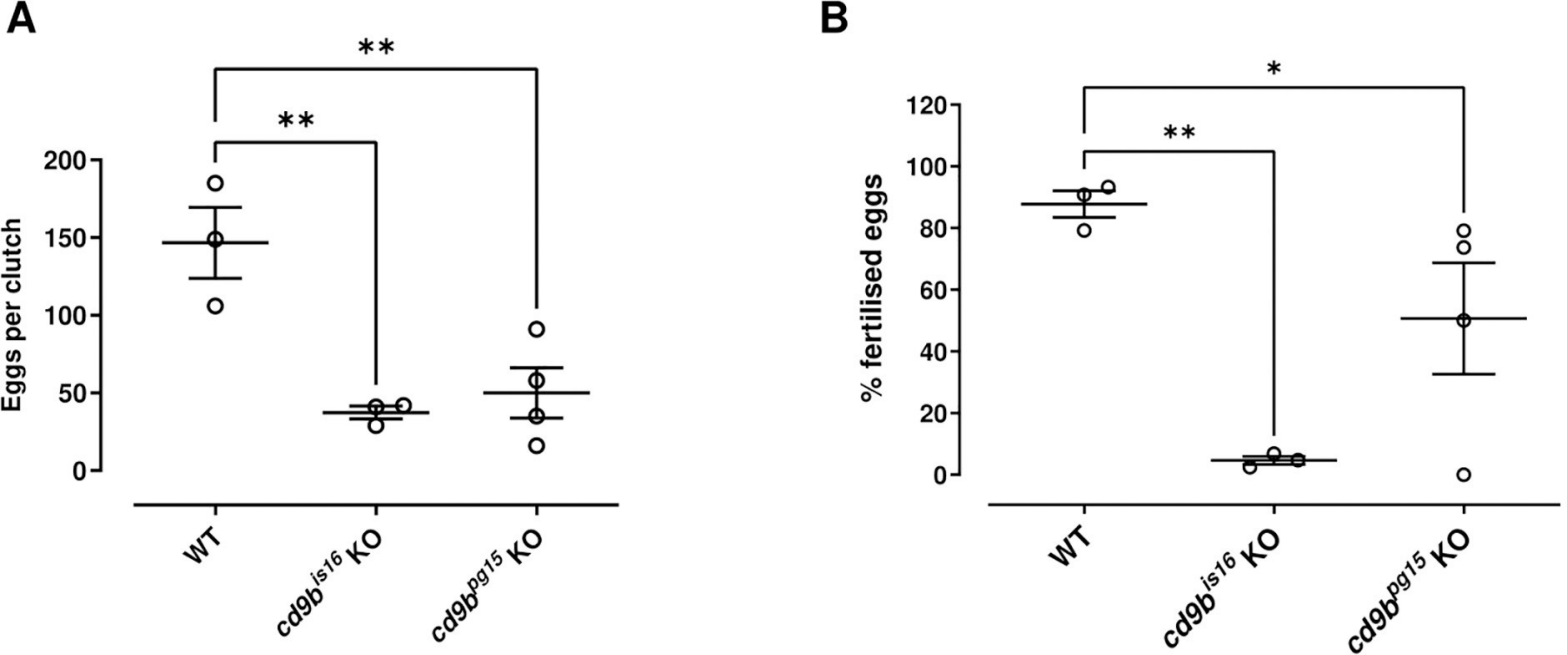
Clutches from *cd9b* mutant pairs, produced by IVF, have a decreased clutch size and lower percentage of eggs fertilised per clutch. The number of eggs is significantly decreased in clutches from *cd9b* mutant pairs (A). Of the eggs laid, a significantly lower percentage were fertilised by *cd9b* mutant males (B). Dead eggs were immediately discarded during the IVF protocol and therefore (A) and (B) represent only fertilised and unfertilised eggs. n = minimum 3 pairs per genotype. ANOVA with Holm-Sidak multiple comparisons test was carried out on the original data from (A) and on arcsine transformed data in (B). The Holm-Sidak post-hoc test was chosen as it has more power than Dunnett’s multiple comparisons test. Significance of difference from WT control: ** p<0.01; * p<0.05; ns = not significant. Data from a single experiment.

It is known that egg release and fertilisation in zebrafish are affected by mating behaviour, as observed previously (reviewed in [25]). To try to exclude this variable, we attempted to fertilise eggs manually using IVF techniques. In the experiment, fish were pair mated overnight by genotype, but the dividers were not removed so the fish were still exposed to the production and sensing of reproductive pheromones required for zebrafish breeding [26, 27]. Fish of the same genotype were then group housed the following morning and individual female fish removed for egg extraction. We found that numbers of eggs obtained from female *cd9b* mutants was significantly lower than WT and similar numbers of eggs were obtained from both mutant alleles (Fig 3A). To assess fertilisation rates, eggs and sperm from the same genotype were mixed externally. Fertilisation rates using sperm from *cd9b* KO males to fertilise KO eggs were also significantly reduced compared to using WT sperm to fertilise WT eggs (Fig 3B). The reduction in the percentage of fertilised eggs is again markedly different between the two alleles, which echoes the difference between the alleles seen in Fig 1B. Overall, this suggests the reductions in clutch size and fertilisation in *cd9b* KO mutants has a non-behavioural element.

The reduction in the number of eggs extracted from *cd9b* mutant females during the IVF protocol might indicate that Cd9b has a role in ovulation, with reduced ovulation induced in *cd9b* mutant females. Female zebrafish are stimulated to ovulate overnight by steroid glucuronides that are produced by the Leydig cells in the testis of male zebrafish, and then released into the water [26–28]. The IVF protocol required fish of the same genotype to be pair mated overnight and so the decreased numbers of eggs extracted from *cd9b* mutant females could be due to an impact on steroid glucuronide production or release in the males, or sensing in the females.

An alternative role for Cd9b in zebrafish fertility could be in gamete fusion. In mice, CD9 has been shown to be required for sperm-egg fusion and for the correct formation and distribution of microvilli on the oolemma [17, 29–32]. The role of CD9 in gamete fusion is suggested to be a result of this regulation of the microvilli [4, 31]. It would therefore be interesting to study the structure of microvilli and sperm-egg binding in the *cd9b* zebrafish mutants in future experiments.

While these preliminary IVF experiments suggest a non-behavioural element in the reduced fecundity and fertilisation seen in *cd9b* KO mutants, the protocol does not eliminate any potential anatomical differences in *cd9b* mutant females that could impede egg laying or investigate possible reductions in sperm production, release, or motility. It would also be useful to investigate these potential mechanisms in future experiments.

**Fig 4:**
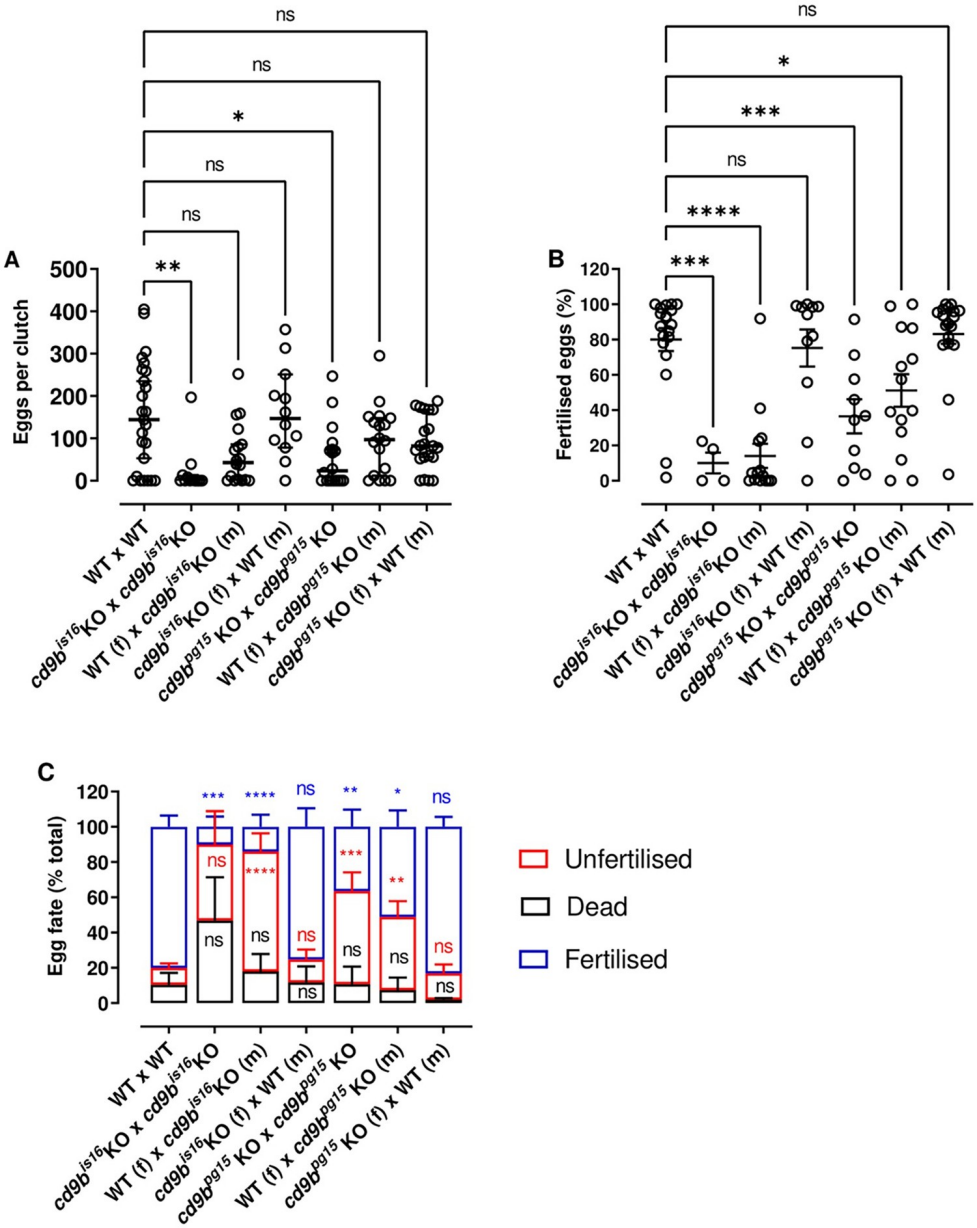
Clutch size can be rescued by substitution of either *cd9b* mutant partner for an WT, however, fertilisation is only rescued when the *cd9b* KO male is substituted for an WT male. Clutch size can be rescued by substitution of a *cd9b* mutant partner, male (m) or female (f), with an WT (A). A Kruskal-Wallis test with Dunn’s multiple comparisons test was carried out on the pooled data from three experimental repeats. n = minimum 12 pairs per cross. While clutch size can be partially or fully rescued by substitution of either *cd9b* mutant partner, the percentage of fertilised eggs appears to only return to WT levels upon substitution of a *cd9b* mutant male with an WT male (B). An ANOVA with Dunnett’s multiple comparisons test was carried out on arcsine transformed data pooled from three experiments. The average percentage of fertilised, dead and unfertilised eggs per clutch is shown in (C). n = minimum 10 pairs per cross for (B) and (C), except *cd9b*^*is16*^ x *cd9b*^*is16*^ where only 4 of 12 pairs laid. Significance of difference from WT control: **** p<0.001; *** p<0.005; ** p<0.01; * p<0.05; ns = not significant. Data was pooled from 3 experimental repeats carried out over several weeks.

To investigate whether the phenotypes were due to a difference in the females, as seen in mice with fertilisation, or due to cumulative effects from both parents, we measured clutch size and fertilisation rates using a matrix of crossings. As found previously, mutant females crossed with mutant males had decreased clutch size and fertilisation rates, with the phenotypes seen in both the *cd9b*^*is16*^ in-crosses and the *cd9b*^*pg15*^ in-crosses (Fig 4A and 4B). Crossing *cd9b* mutants of either gender with WT fish produced normal clutch sizes (Fig 4A), showing that this phenotype can be rescued by both male and female WT fish. This data shows that *cd9b* mutant females have the ability to ovulate and lay normal numbers of eggs, which suggests that the decrease in clutch size seen with *cd9b* mutant in-crosses is not due to potential anatomical differences in the *cd9b* mutant female that could impeding egg laying. Indeed, given that clutch size can be rescued by replacing a *cd9b* mutant of either gender with a WT, this data suggests that both genders have a role in this phenotype.

The requirement of reproductive pheromones for successful zebrafish breeding could be a possible explanation for the role of both genders in the decrease in clutch size seen from *cd9b* mutant pairs [26, 27]. As mentioned above, female zebrafish ovulate in response to steroid glucuronides released into the water by male zebrafish. Similarly, female zebrafish produce and secrete steroid glucuronides, such as oestradiol-17β-glucuronide and testosterone-glucuronide, which then attract and initiate courtship behaviour in the male zebrafish to facilitate egg laying [26–33]. It would therefore be interesting to investigate the production, release and sensing of steroid glucuronides by *cd9b* mutants and analyse whether the mutants display any differences in courtship behaviour (e.g. chasing, contact using the nose or tail, approaching, encircling and presenting etc) [34].

In contrast to clutch size, the defect in the percentage of eggs fertilised was only rescued when the *cd9b* mutant male was substituted for a WT male (Fig 4B and 4C). This suggests that the reduction in fertilisation seen in *cd9b* mutant pairs is due solely to a difference in the mutant male, which is the opposite to that seen in CD9b KO mice, where CD9 is required for female fertility [17, 29, 30]. Given that the reduction in fertility in *cd9b* mutants appears to be due to a defect in the mutant male, it would be interesting to investigate whether Cd9b plays a role in sperm release, sperm motility or sperm—egg binding. A reduced quality or quantity of sperm could also result in the reduced fertility seen and so future work could include investigating if the *cd9b* mutation has an impact on spermatogenesis. This could include looking at the steroid hormones that control spermatogenesis, histological examinations of spermatogenesis in the testis and conducting sperm counts. Furthermore, CD9 is expressed throughout the majority of murine spermatogenesis and it would be interesting to investigate whether this expression is replicated in zebrafish [6, 23, 24].

Although there is no statistically significant difference in the number of dead eggs observed between the two alleles, an increased trend can be seen for *cd9b*^*is16*^(Fig 4C). It would be beneficial to undertake future work to study the egg fate in *cd9b*^*is16*^ fish in more detail. This would include determining if the phenotype can be rescued and, given that eggs were collected every 20 minutes and dead eggs counted and removed on collection, it would also be interesting to determine if the eggs are laid dead or die shortly after laying.

In conclusion, as with CD9 KO mice, *cd9b* homozygous mutant zebrafish showed fertility defects. It was found that *cd9b* KO zebrafish pairs laid decreased numbers of eggs and *cd9b* KO males had severely reduced fertility. In mice and human, CD9 appears to facilitate sperm penetration of the oolemma rather than the initial binding to the plasma membrane [35, 36]. CD9 KO female mice display a severe reduction in fertility due to defective sperm-egg fusion, but show no ovulation defects [17, 29, 30]. It was therefore surprising that the *cd9b* zebrafish mutants laid significantly fewer eggs and that the fertility phenotype appeared to be due to a defect in the *cd9b* mutant male, unlike CD9 KO mice. Unlike the mammal, it appears that CD9 plays a more complex role in fertility in zebrafish involving both sperm and oocyte. CD9 has, however, been reported to be expressed in male mice throughout spermatogenesis and in mature sperm during fertilisation [6, 23]. It would be interesting to investigate whether *cd9b* is similarly expressed in zebrafish males and to undertake further studies to elucidate the underlying mechanism behind the fertility phenotype. Perhaps infertility studies would benefit from CD9 investigation, an understudied membrane protein in regards to human fertility, in men in particular.

## Supporting information

S1 Fig *cd9b* mutant generation. A: Nature of the *cd9b* mutant allele showing TALEN site location within the intron-exon structure of the gene. B: The TALEN target sequence in exon 1 is shown in blue; the 8bp deletion in the *cd9b*^*pg15*^ allele, or the 1bp deletion in the *cd9b*^*is16*^ allele is indicated under the WT sequence as dashes. The 8bp deletion leads to a frameshift changing codon 15 from TTT (Phe) to CAA (Glu), then 22 aberrant amino acids (red lettering) followed by a stop codon (*). The 1bp deletion leads to a frameshift changing codon 16 from ATC (Ile) to TCT (Ser), then 46 aberrant amino acids (red lettering) followed by a stop codon (*). C: Schematic of the Cd9b protein with location of mutation given by red arrow. The disulfide bonds between the conserved CCG motif and conserved cysteines are indicated by the dashed lines. EC1/2 = Extracellular domain 1/2, aa = amino acid. D-F: Sequence chromatograms of genomic DNA from (d) WT and (e) *cd9b*^*pg15*^ alleles and (f) *cd9b*^*is16*^ alleles. Location of mutation is underlined in red.
(DOCX)

S2 Fig qPCR shows *cd9b* is significantly decreased in *cd9b* KO embryos. A. Expression of *cd9b* is significantly reduced in both *cd9b* mutants compared to AB embryos. qPCR on single 36 hpf embryo cDNA using 6 biological samples and three technical repeats for each condition. Abnormal results, due to pipetting errors, were removed. Unpaired T-test with Holm-Sidak’s multiple comparisons correction, p = <0.05. n = minimum 15 data points per genotype. B-C: Dissociation curves of (b) β-actin 2 and (c) *cd9b* show the qPCR primer pairs produce a single product. n = 4 technical repeats.
(DOCX)

S3 Fig WISH shows *cd9b* is significantly decreased in *cd9b* KO embryos. A-C: Representative images of *cd9b* WISH at 36 hpf in (a) WT, (b) *cd9b*^*pg15*^ homozygous embryos and (c) *cd9b*^*is16*^ homozygous embryos. (a) *cd9b* can be seen in the neuromasts and primordium of the posterior lateral line in WT embryos (arrows), but is absent in *cd9b* mutants (b,c). n = minimum 3 imaged, 10 observed per genotype. Data from a single experiment.
(DOCX)
