# Supplementary figures and images for: Tetraspanin Cd9b plays a role in fertility in zebrafish
Source: PLoS One. 2022 Nov 10;17(11):e0277274. doi: 10.1371/journal.pone.0277274 (PMC9648739; doi:10.1371/journal.pone.0277274)

**
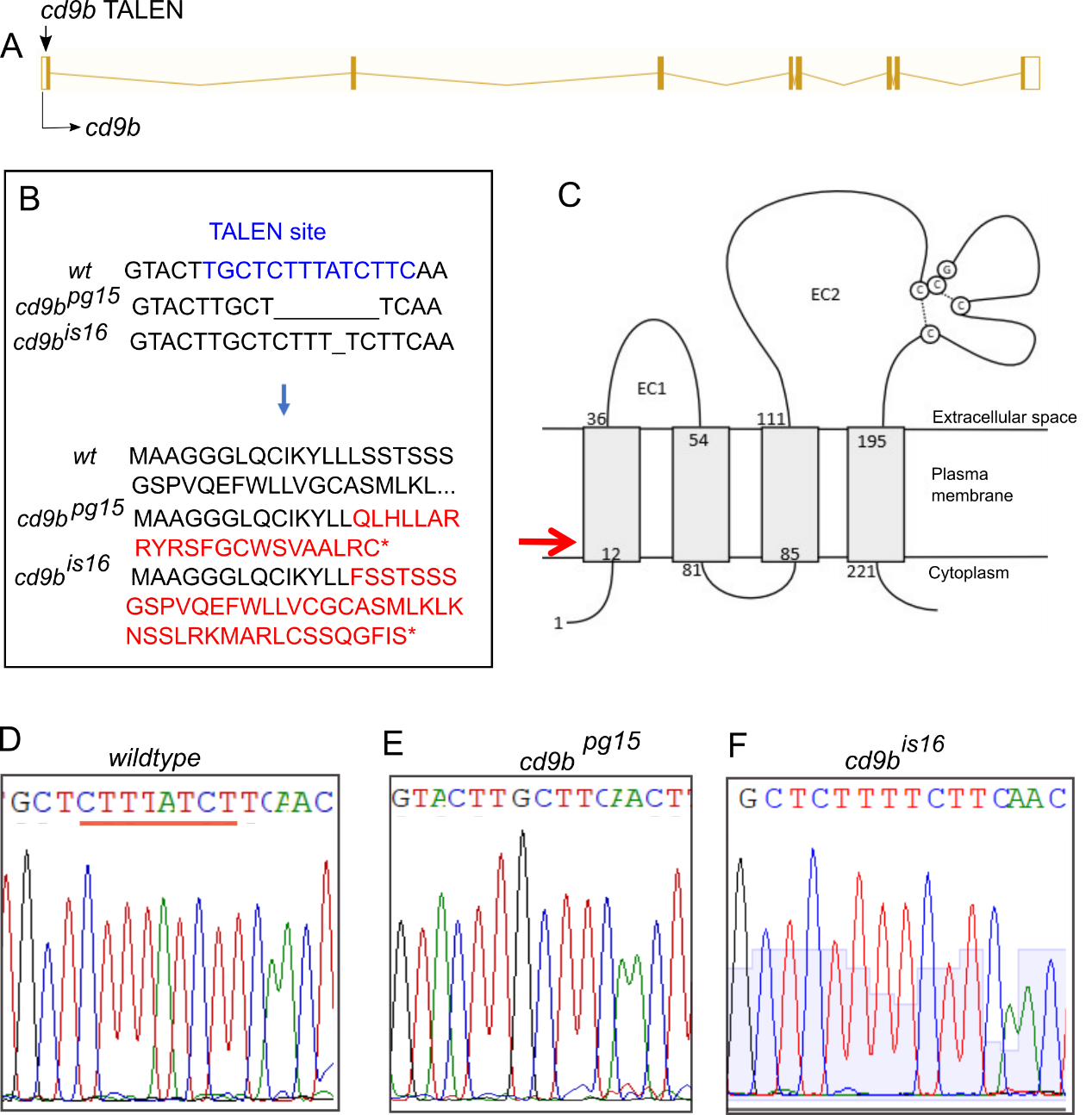
Supplementary Figure 1. *cd9b* mutant generation.**

Supplement: S1 Fig — A: Nature of the cd9b mutant allele showing TALEN site location within the intron-exon structure of the gene. B: The TALEN target sequence in exon 1 is shown in blue; the 8bp deletion in the cd9bpg15 allele, or the 1bp deletion in the cd9bis16 allele is indicated under the WT sequence as dashes. The 8bp deletion leads to a frameshift changing codon 15 from TTT (Phe) to CAA (Glu), then 22 aberrant amino acids (red lettering) followed by a stop codon (*). The 1bp deletion leads to a frameshift changing codon 16 from ATC (Ile) to TCT (Ser), then 46 aberrant amino acids (red lettering) followed by a stop codon (*). C: Schematic of the Cd9b protein with location of mutation given by red arrow. The disulfide bonds between the conserved CCG motif and conserved cysteines are indicated by the dashed lines. EC1/2 = Extracellular domain 1/2, aa = amino acid. D-F: Sequence chromatograms of genomic DNA from (d) WT and (e) cd9bpg15 alleles and (f) cd9bis16 alleles. Location of mutation is underlined in red. (DOCX) [file pone.0277274.s001.docx]

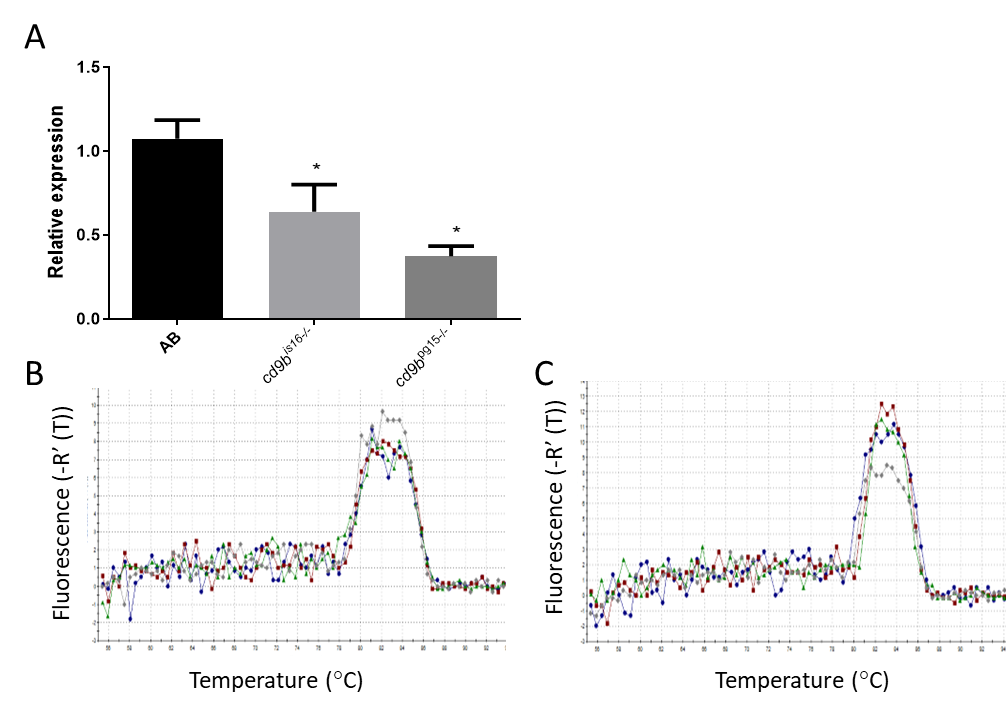


**Supplementary Figure 2. qPCR shows *cd9b* is significantly decreased in *cd9b* KO embryos.**

Supplement: S2 Fig — A. Expression of cd9b is significantly reduced in both cd9b mutants compared to AB embryos. qPCR on single 36 hpf embryo cDNA using 6 biological samples and three technical repeats for each condition. Abnormal results, due to pipetting errors, were removed. Unpaired T-test with Holm-Sidak’s multiple comparisons correction, p = <0.05. n = minimum 15 data points per genotype. B-C: Dissociation curves of (b) β-actin 2 and (c) cd9b show the qPCR primer pairs produce a single product. n = 4 technical repeats. (DOCX) [file pone.0277274.s002.docx]

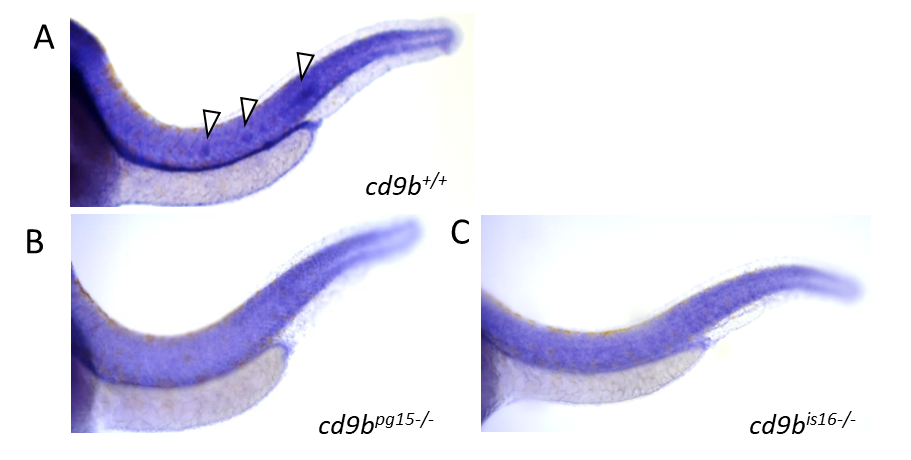
**Supplementary Figure 3. WISH shows *cd9b* is significantly decreased in *cd9b* KO embryos.**

Supplement: S3 Fig — A-C: Representative images of cd9b WISH at 36 hpf in (a) WT, (b) cd9bpg15 homozygous embryos and (c) cd9bis16 homozygous embryos. (a) cd9b can be seen in the neuromasts and primordium of the posterior lateral line in WT embryos (arrows), but is absent in cd9b mutants (b,c). n = minimum 3 imaged, 10 observed per genotype. Data from a single experiment. (DOCX) [file pone.0277274.s003.docx]
